# Establishing an artificial intelligence-based predictive model for long-term health-related quality of life for infected patients in the ICU

**DOI:** 10.1016/j.heliyon.2024.e35521

**Published:** 2024-07-31

**Authors:** Yang Zhang, Sinong Pan, Yan Hu, Bingrui Ling, Tianfeng Hua, Lunxian Tang, Min Yang

**Affiliations:** a The Second Department of Critical Care Medicine, The Second Affiliated Hospital of Anhui Medical University, Hefei, Anhui, 230601, PR China; b Laboratory of Cardiopulmonary Resuscitation and Critical Care, The Second Affiliated Hospital of Anhui Medical University, Hefei, Anhui, 230601, PR China; c Department of Internal Emergency Medicine (North), Shanghai East Hospital, Tongji University School of Medicine, Shanghai, 200120, PR China

**Keywords:** Infection, Sepsis, Health-related quality of life, Prognostic model, XGBoost, Artificial intelligence

## Abstract

**Objective:**

To develop a model using a Chinese ICU infection patient database to predict long-term health-related quality of life (HRQOL) in survivors.

**Methods:**

A patient database from the ICU of the Fourth People's Hospital in Zigong was analyzed, including data from 2019 to 2020. The subjects of the study were ICU infection survivors, and their post-discharge HRQOL was assessed through the SF-36 survey. The primary outcomes were the physical component summary (PCS) and mental component summary (MCS). We used artificial intelligence techniques for both feature selection and model building. Least absolute shrinkage and selection operator regression was used for feature selection, extreme gradient boosting (XGBoost) was used for model building, and the area under the receiver operating characteristic curve (AUROC) was used to assess model performance.

**Results:**

The study included 917 ICU infection survivors. The median follow-up was 507.8 days. Their SF-36 scores, including PCS and MCS, were below the national average. The final prognostic model showed an AUROC of 0.72 for PCS and 0.63 for MCS. Within the sepsis subgroup, the predictive model AUROC values for PCS and MCS were 0.76 and 0.68, respectively.

**Conclusions:**

This study established a valuable prognostic model using artificial intelligence to predict long-term HRQOL in ICU infection patients, which supports clinical decision making, but requires further optimization and validation.

## 1 Introduction

Worldwide, infections are a common challenge in intensive care units (ICUs), with significant morbidity and mortality [1]. An epidemiologic study involving 13,796 patients across 1,265 ICUs in 75 countries found that 51 % of patients were diagnosed with infections, and 71 % received antibiotic therapy [2]. Sepsis, characterized by dysregulated inflammatory responses leading to organ dysfunction, has an incidence rate of approximately 39 % in infected patients, a proportion that is more pronounced in the ICU [[3], [4], [5]]. Furthermore, sepsis is the leading cause of mortality in these patients. Statistically, an estimated 48 million people worldwide develop sepsis annually, resulting in approximately 11 million deaths [6,7]. Notably, China is one of the countries experiencing the most rapid increase in sepsis-related mortality, affecting one-fifth of its mainland ICU population [8].

Over the past few decades, significant progress has been made in the early detection and treatment of sepsis, which has significantly improved the short-term survival rates associated with this condition [9]. However, as the number of sepsis survivors escalates, the subsequent impact on their long-term health-related quality of life (HRQOL) has received increasing attention from both the medical community and society at large [10,11]. Data show a generalized decline in HRQOL among sepsis survivors, including limitations in physiological functionality, increased psychological distress, and changes in societal roles [[12], [13], [14], [15], [16]]. This issue is similarly manifested among sepsis survivors in China [17,18]. Such circumstances not only impose ongoing psychological and existential burdens on patients and their families, but also present novel challenges for the long-term allocation and management of medical resources [[19], [20], [21], [22]].

The long-term HRQOL of ICU patients who have survived infections and sepsis is an emerging area of research that is receiving increasing attention. However, existing studies predominantly emphasize short-term treatments and mortality reduction, with insufficient focus on the long-term HRQOL of these individuals, and predictive models are conspicuously absent. Therefore, our study, based on a database of Chinese ICU patients with infections, aims to develop a model to predict the long-term HRQOL of these individuals. At the same time, we endeavor to analyze and elucidate factors that may influence patients' HRQOL, thereby providing guidance for medical interventions.

## 2 Methods

### 2.1 Data source

This study was derived from the database of the Fourth People's Hospital in Zigong City, Sichuan Province, China, which includes data of ICU patients diagnosed with infections between January 2019 and December 2020 [23,24]. The database provides comprehensive details for each patient, including length of stay, vital signs, laboratory tests, pharmacological treatments, and follow-up information. Follow-up data were collected through telephone interviews conducted by professional staff with the patients or their family members to complete the SF-36 questionnaire. The follow-up period ranged from over one year to less than three years after hospital admission. To ensure patient confidentiality, all personal identifiers were de-identified and replaced with random codes, eliminating the need for informed consent. The researcher (Zhang) has completed the training program provided by the collaborating institution (Certificate No. 53496787) and is qualified to use the database and extract data. This study adheres to the transparent reporting of a multivariate prediction model for individual prognosis or diagnosis (TRIPOD) statement guidelines.

### 2.2 Study population

All ICU patients with infections in the database were included. Exclusions included: (1) patients younger than 18 years; (2) patients who died during hospitalization or before follow-up; and (3) patients lost to follow-up.

### 2.3 Definition of infection

Infection was defined according to the original database descriptions, which identified infections based on the presence of keywords such as **“**infection”, “pneumonia” and various forms of “-itis” in the diagnosis**.** The original diagnosis descriptions, recorded in simplified Chinese, included terms like “*Ganran*” and “*Yan*” to pinpoint relevant cases. Autoimmune or connective tissue diseases were manually excluded from the infection category to maintain the specificity of the infection classification [23,24].

### 2.4 Outcome variables

The outcome measure for the study was post-discharge HRQOL as assessed by the SF-36 questionnaire, a predominant tool for assessing HRQOL in ICU survivors and validated for use in sepsis survivors (Supplementary file 1) [25,26]. The SF-36 questionnaire consists of 36 items that are further subdivided into eight domains: physical functioning (PF), role-functioning physical (RP), bodily pain (BP), general health (GH), vitality (VT), social functioning (SF), role-functioning emotional (RE), and mental health (MH). Scores for each domain range from 0 to 100, with lower scores indicating greater impairment or disability in that domain [27]. Composite scores for the physical component summary (PCS) and mental component summary (MCS) can be calculated from the scores of these eight domains. Lower PCS and MCS scores indicate more severe physical or mental impairment, compared to a general Chinese population mean score of 50 for both PCS and MCS [28].

In this study, the outcome variables were PCS and MCS, and the scores were calculated according to the standard methodology for the Chinese normative sample [28]. For subsequent analyses, both outcome variables were dichotomized: based on the general Chinese population benchmark, scores of PCS or MCS ≥50 were classified as "good" HRQOL, while scores <50 were classified as "poor" HRQOL.

### 2.5 Additional variables

Other variables included: (1) demographics; (2) admission department, discharge department, initial ICU admission type, and infection classification; (3) vital signs and preliminary laboratory values within the first 24 h after ICU admission; (4) sequential organ failure assessment (SOFA) and Glasgow coma scale (GCS) scores; (5) comorbidities, including hypertension, diabetes mellitus, congestive heart failure, chronic obstructive pulmonary disease, chronic kidney disease, stroke, intracerebral hemorrhage, and traumatic brain injury; (6) tracheostomy status, mechanical ventilation, vasopressor administration, analgesic and sedative use; and (7) hospital length of stay and ICU length of stay.

### 2.6 Data processing

Variables with missing values exceeding 20 % were excluded. For continuous variables, outlying and conspicuously inconsistent values were treated as missing data. Multiple imputation for missing values was performed using the "MICE" package [29]. One-hot encoding was used to represent unordered multi-categorical variables.

### 2.7 Statistical analysis

Continuous variables were presented as median with interquartile range and compared using the Mann-Whitney *U* test. Categorical variables were presented as frequencies and percentages, with comparisons made using the chi-squared test or Fisher's exact test.

The final dataset was randomly divided into training and validation sets in an 8:2 ratio. Dimensionality reduction and feature selection were conducted using the least absolute shrinkage and selection operator (LASSO) regression, a common artificial intelligence (AI) technique to improve model accuracy and interpretability. Subsequently, the extreme gradient boosting (XGBoost) approach, an advanced AI and machine learning technique, was used to construct predictive models for the PCS and MCS. XGBoost is a tree-based ensemble machine learning method that optimizes predictive results by constructing multiple decision trees and employing a gradient boosting strategy. During the construction of each tree, the cumulative errors of all previous trees are considered. By optimizing an objective function that includes a regularization term, XGBoost reduces overfitting and enhances the model's generalization ability. In addition, XGBoost supports parallel processing of features, improving processing speed and efficiency [30].

The primary performance metric for the final model evaluation was the area under the receiver operating characteristic curve (AUROC). Additional metrics included sensitivity, specificity, accuracy, precision, and F1 score. Shapley additive explanations (SHAP) values were used to explore the interpretability of the final predictive model, with a ranking of predictor importance based on SHAP values [31]. In addition, based on a criterion of SOFA score ≥2, a subgroup analysis of enrolled sepsis patients was performed and XGBoost was applied to build predictive models for PCS and MCS in this subgroup. All statistical analyses were performed using R version 4.2.3 and STATA version 15.1, with a *P* value < 0.05 considered statistically significant.

## 3 Results

### 3.1 Participants and baseline characteristics

**Fig. 1:**
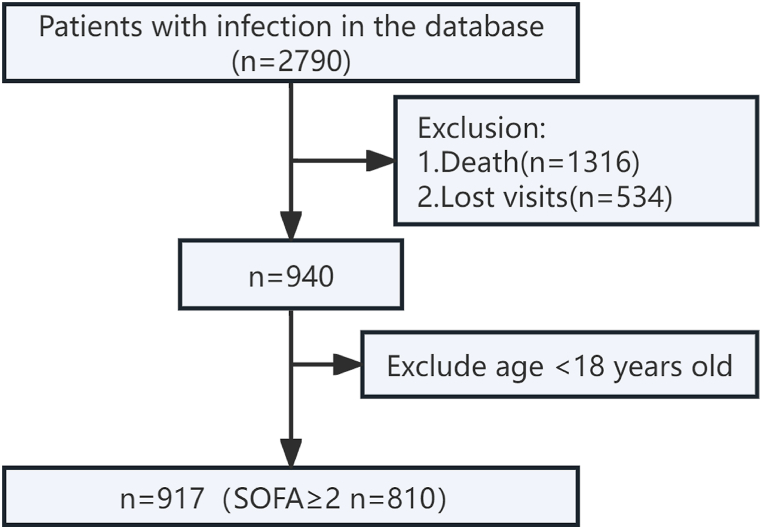
Flowchart. Abbreviations: SOFA sequential organ failure assessment*.*

**Fig. 2:**
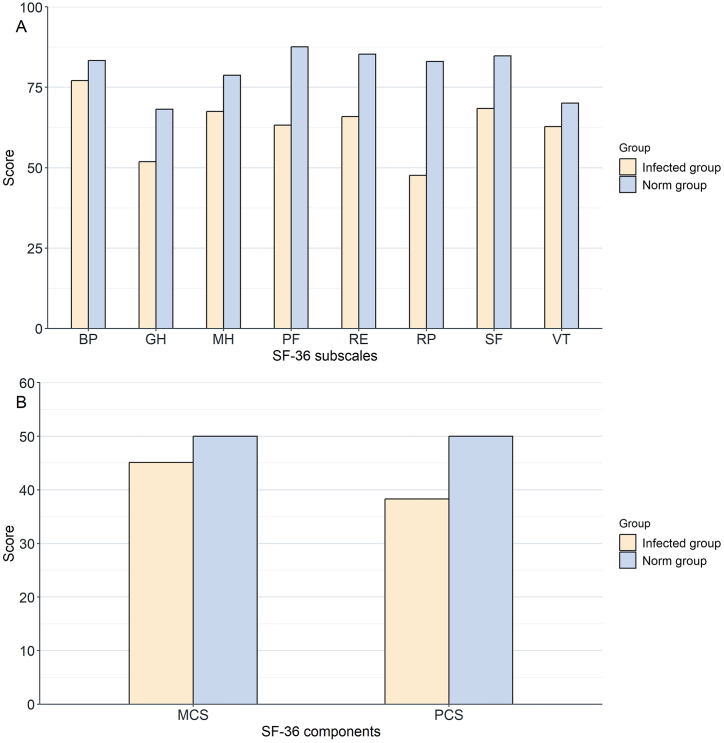
A. Comparison of mean scores for the eight dimensions of the SF-36 scale between ICU infection survivors and Chinese standard norms; B. Comparison of mean MCS and PCS scores between ICU infection survivors and Chinese standard norms. Abbreviations: PF physical functioning, RP role-functioning physical, BP bodily pain, GH general health, VT vitality; SF social functioning, RE role-functioning emotional, MH mental health, PCS physical component summary, MCS mental component summary.

A total of 2,790 ICU patients with infections were identified from the database. According to the exclusion criteria, 917 patients were finally included in the analysis (Fig. 1). Table 1 describes the baseline characteristics of all enrolled patients and compares the "good" and "poor" groups according to their MCS and PCS scores. The median follow-up for the entire cohort was 507.8 days. Across the eight domains of the SF-36 scale, the scores of ICU infection survivors were generally lower than those of the Chinese general population (Fig. 2A). Fig. 2B suggests that ICU infection survivors had lower PCS and MCS scores compared with the Chinese general population.

**Table 1:** Baseline characteristics.

Variables	Total (*n* = 917)	PCS <50 poor (*n* = 578)	PCS ≥50 good (*n* = 339)	*P*	MCS <50 poor (*n* = 441)	MCS ≥50 good (*n* = 476)	*P*
Age	65 (52, 74)	67 (55, 77)	59 (46.5, 71)	<0.01	65 (53, 75)	64 (51, 74)	0.14
**Sex, n (%)**				0.78			0.74
Female	391 (42.6)	249 (43.1)	142 (41.9)		391 (42.6)	191 (43.3)	
Male	526 (57.4)	329 (56.9)	197 (58.1)		526 (57.4)	250 (56.7)	
**Admission department, n (%)**				<0.01			0.03
ICU	136 (14.8)	93 (16.1)	43 (12.7)		74 (16.8)	62 (13)	
Medicine	351 (38.3)	245 (42.4)	106 (31.3)		174 (39.5)	177 (37.2)	
Surgical	381 (41.5)	225 (38.9)	156 (46)		178 (40.4)	203 (42.6)	
Other	49 (5.3)	15 (2.6)	34 (10)		15 (3.4)	34 (7.1)	
**Discharge department, n (%)**				<0.01			0.03
ICU	136 (14.8)	93 (16.1)	43 (12.7)		74 (16.8)	62 (13)	
Medicine	351 (38.3)	245 (42.4)	106 (31.3)		174 (39.5)	177 (37.2)	
Surgical	381 (41.5)	225 (38.9)	156 (46)		178 (40.4)	203 (42.6)	
Other	49 (5.3)	15 (2.6)	34 (10)		15 (3.4)	34 (7.1)	
**First ICU, n (%)**				0.01			0.98
EICU	381 (41.5)	231 (40)	150 (44.2)		184 (41.7)	197 (41.4)	
ICU	518 (56.5)	330 (57.1)	188 (55.5)		248 (56.2)	270 (56.7)	
Other	18 (2)	17 (2.9)	1 (0.3)		9 (2)	9 (1.9)	
**Infection site, n (%)**				<0.01			0.19
Abdomen	83 (9.1)	47 (8.3)	36 (10.3)		35 (8.4)	48 (9.6)	
Brain	8 (0.9)	4 (0.7)	4 (1.1)		5 (1.2)	3 (0.6)	
Digestive	95 (10.4)	42 (7.4)	53 (15.1)		32 (7.7)	63 (12.6)	
Pneumonia	554 (60.4)	360 (63.6)	194 (55.3)		259 (62.1)	295 (59)	
Soft tissue	27 (2.9)	15 (2.7)	12 (3.4)		11 (2.6)	16 (3.2)	
Urine	55 (6)	36 (6.4)	19 (5.4)		29 (7)	26 (5.2)	
Others	95 (10.4)	62 (11)	33 (9.4)		46 (11)	49 (9.8)	
**Vital signs**							
Heart rate, (min^−1^)	86 (75, 98)	86 (76, 99)	84 (74, 96.5)	0.09	86 (76, 98)	85.5 (74, 97)	0.34
Temperature, (°C)	36.7 (36.5, 37)	36.7 (36.5, 37)	36.7 (36.5, 37.1)	0.10	36.7 (36.5, 37)	36.7 (36.5, 37)	0.22
Respiratory rate, (min^−1^)	19 (17, 21)	19 (18, 21)	19 (17, 21)	0.13	19 (18, 21)	19 (17, 21)	0.85
Spo_2_, (%)	99 (98, 100)	99 (98, 100)	99 (98, 100)	0.55	99 (98, 100)	99 (98, 100)	0.32
Systolic BP, (mmHg)	125 (111, 140)	127.5 (113, 143)	123 (109, 137)	<0.01	128 (112, 142)	124 (110, 138)	0.01
Diastolic BP, (mmHg)	71 (63, 80)	71 (63, 81)	71 (62, 80)	0.46	72 (62, 81)	71 (63, 80)	0.78
**Laboratory tests**							
Hemoglobin, (g/L)	107 (92, 123)	108 (94, 123)	105 (91, 121.5)	0.10	108 (94, 123)	107 (91, 122.2)	0.34
Albumin, (g/L)	32.7 (29.4, 36.4)	32.5 (29.3, 35.9)	33.2 (29.6, 37.1)	0.05	32.6 (29.6, 36)	32.8 (29.4, 36.6)	0.89
Creatinine, (umol/L)	54.8 (44.3, 69.7)	56.8 (45.3, 72.1)	52.7 (43.4, 67.1)	0.01	55.3 (44.8, 71.4)	54.2 (43.7, 69)	0.26
BUN, (mmol/L)	5.4 (3.9, 7.6)	5.7 (4.1, 7.9)	4.9 (3.5, 7.2)	<0.01	5.4 (3.8, 7.5)	5.4 (4, 7.7)	0.76
Total bilirubin, (umol/L)	11.2 (8, 16.7)	11.3 (8, 16.8)	11.2 (8, 16.6)	0.66	11 (7.9, 16.2)	11.4 (8, 17.5)	0.20
Glucose, (mmol/L)	6.2 (5.2, 8.4)	6.2 (5.2, 8.3)	6.4 (5.3, 8.6)	0.60	6.1 (5.2, 8)	6.4 (5.3, 8.7)	0.26
BNP, (pg/mL)	81.9 (32.5, 212.8)	92.4 (33, 222)	71.8 (31.4, 190.2)	0.06	86.8 (33.2, 230.3)	79.9 (32.3, 201)	0.35
Anion gap, (mmol/L)	10.3 (8.4, 12.4)	10.3 (8.3, 12.8)	10.3 (8.5, 12.1)	0.85	10.3 (8.2, 12.6)	10.3 (8.5, 12.3)	0.63
Sodium, (mmol/L)	138.8 (136.2, 140.9)	138.7 (136, 140.9)	138.8 (136.6, 140.9)	0.42	139 (136.4, 141.1)	138.6 (136, 140.8)	0.17
Chloride, (mmol/L)	101.9 (98.8, 104.5)	101.5 (98.5, 104.5)	102.3 (99.3, 104.6)	0.08	101.8 (98.8, 104.7)	101.9 (99, 104.5)	0.81
Potassium, (mmol/L)	4 (3.6, 4.3)	3.9 (3.6, 4.3)	4 (3.7, 4.3)	0.53	4 (3.6, 4.3)	4 (3.6, 4.3)	0.59
Magnesium, (mmol/L)	0.8 (0.7, 0.9)	0.8 (0.7, 0.9)	0.8 (0.8, 0.9)	0.97	0.8 (0.7, 0.9)	0.8 (0.8, 0.9)	0.93
Total calcium, (mmol/L)	2.2 (2.1, 2.3)	2.2 (2.1, 2.3)	2.2 (2, 2.3)	0.44	2.2 (2.1, 2.3)	2.2 (2, 2.3)	0.07
Phosphate, (mmol/L)	0.9 (0.8, 1.1)	0.9 (0.8, 1.1)	0.9 (0.8, 1.1)	0.70	0.9 (0.7, 1.1)	0.9 (0.8, 1.1)	0.11
Prothrombin time, (sec)	13.7 (13, 14.6)	13.7 (13, 14.6)	13.6 (13, 14.6)	0.69	13.7 (13, 14.5)	13.7 (13, 14.6)	0.56
APTT, (sec)	30.2 (26.9, 34)	29.8 (26.9, 33.6)	31.2 (27.3, 34.5)	0.01	29.6 (26.8, 33.8)	30.5 (27.3, 34.1)	0.06
INR	1.2 (1.1, 1.3)	1.2 (1.1, 1.2)	1.2 (1.1, 1.3)	0.29	1.2 (1.1, 1.2)	1.2 (1.1, 1.3)	0.03
D-Dimer, (mg/L)	2.9 (1.4, 6.3)	2.8 (1.4, 6)	3.3 (1.6, 6.6)	0.10	2.7 (1.4, 6)	3.1 (1.6, 6.6)	0.12
pH	7.4 (7.4, 7.5)	7.4 (7.4, 7.5)	7.4 (7.4, 7.5)	0.26	7.4 (7.4, 7.5)	7.4 (7.4, 7.5)	0.64
PCO_2_, (mmHg)	39 (34.4, 43)	39 (35, 43)	38.9 (34, 43)	0.21	39 (35, 44)	38.8 (34, 43)	0.12
Bicarbonate, (mmol/L)	25 (22.4, 27.8)	25.1 (22.6, 28.1)	24.7 (22.1, 27.1)	0.06	25.2 (22.4, 28.1)	24.6 (22.3, 27.3)	0.12
Base excess, (mmol/L)	0.8 (−1.8, 3.2)	1 (−1.7, 3.6)	0.5 (−2, 2.8)	0.07	0.9 (−1.7, 3.5)	0.7 (−1.9, 2.9)	0.17
Lactic acid, (mmol/L)	1.5 (1.1, 1.9)	1.5 (1.1, 1.9)	1.5 (1.1, 2)	0.45	1.5 (1.1, 1.9)	1.5 (1.1, 1.9)	0.82
**Score**							
GCS	14 (8, 14)	13 (7, 14)	14 (10, 14)	<0.01	13 (7, 14)	14 (9, 14)	<0.01
SOFA	4 (2, 6)	4 (2, 5)	4 (2, 6)	0.68	4 (2, 5)	4 (2, 6)	0.03
**Comorbidity**							
CHF, n (%)	125 (13.6)	92 (15.9)	33 (9.7)	0.01	69 (15.6)	56 (11.8)	0.11
CKD, n (%)	52 (5.7)	44 (7.6)	8 (2.4)	<0.01	34 (7.7)	18 (3.8)	0.02
COPD, n (%)	105 (11.5)	82 (14.2)	23 (6.8)	<0.01	49 (11.1)	56 (11.8)	0.84
Diabetes, n (%)	167 (18.2)	113 (19.6)	54 (15.9)	0.20	90 (20.4)	77 (16.2)	0.12
Hypertension, n (%)	304 (33.2)	215 (37.2)	89 (26.3)	<0.01	168 (38.1)	136 (28.6)	<0.01
TBI, n (%)	138 (15)	95 (16.4)	43 (12.7)	0.15	77 (17.5)	61 (12.8)	0.06
ICH, n (%)	158 (17.2)	119 (20.6)	39 (11.5)	<0.01	101 (22.9)	57 (12)	<0.01
Cerebral infarction, n (%)	152 (16.6)	120 (20.8)	32 (9.4)	<0.01	90 (20.4)	62 (13)	<0.01
**Treatment measures**							
Vasopressor over 7d, n (%)	65 (7.1)	46 (8)	19 (5.6)	0.23	34 (7.7)	31 (6.5)	0.56
Sedation over 7d, n (%)	238 (26)	172 (29.8)	66 (19.5)	<0.01	137 (31.1)	101 (21.2)	<0.01
Analgesia over 7d, n (%)	223 (24.3)	159 (27.5)	64 (18.9)	<0.01	128 (29)	95 (20)	<0.01
MV over 7d, n (%)	165 (18)	128 (22.1)	37 (10.9)	<0.01	99 (22.4)	66 (13.9)	<0.01
Tracheotomy, n (%)	43 (4.7)	35 (6.1)	8 (2.4)	0.02	27 (6.1)	16 (3.4)	0.07
**Events**							
ICU stay, (hour)	86.6 (44.3, 199.4)	94.6 (46.7, 233.9)	71.6 (38.1, 140.1)	<0.01	94.4 (48, 243.9)	79.8 (39.1, 157.5)	<0.01
Hospital stay, (hour)	439.9 (259.7, 793.9)	452.2 (275.7, 1041.8)	435.2 (231.7, 654.1)	<0.01	489.4 (299.9, 1162.4)	409.4 (223, 645.5)	<0.01

Abbreviations: PCS physical component summary, MCS mental component summary, ICU intensive care unit, EICU emergency intensive care unit, BP blood pressure, BUN blood urea nitrogen, BNP b-type natriuretic peptide, APTT activated partial thromboplastin time, INR international normalized ratio, GCS Glasgow coma scale, SOFA sequential organ failure assessment, CHF congestive heart failure, CKD chronic kidney disease, COPD chronic obstructive pulmonary disease, TBI traumatic brain injury, ICH intracerebral hemorrhage, MV mechanical ventilation.

### 3.2 Feature selection

Prior to modeling for PCS and MCS, variables were selected, initially identifying 52 feature variables (Table 1). After one-hot encoding of unordered multi-categorical variables, this was expanded to 66 feature variables. Subsequent LASSO regression, using lambda 1se as the selection criterion, reduced the PCS predictive model to 23 variables of significant predictive ability and the MCS predictive model to only 4 salient predictive variables (Supplementary file 2 Table S1).

### 3.3 Model development, performance, and interpretation

**Fig. 3:**
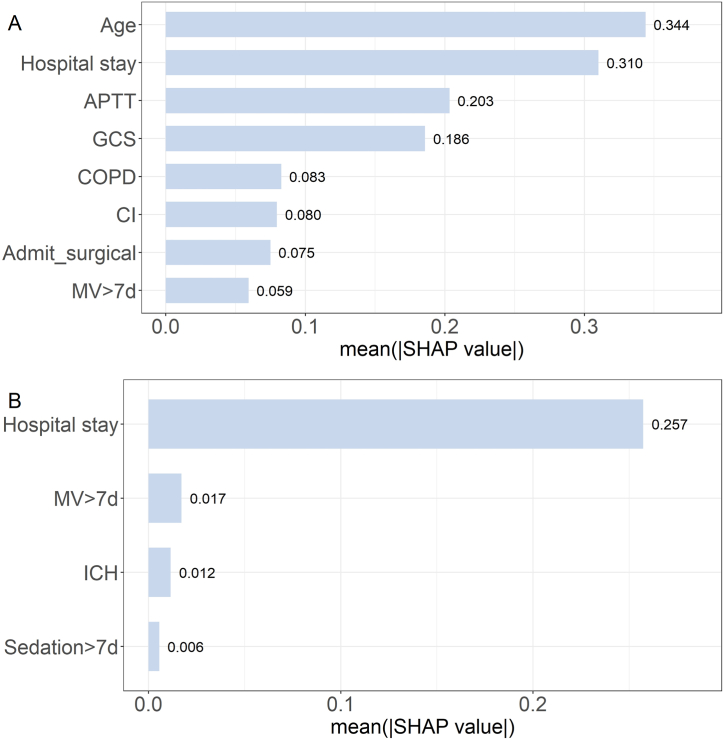
A. Variable importance ranking for the PCS prediction model; B. Variable importance ranking for the MCS prediction model. Abbreviations: PCS physical component summary, MCS mental component summary, APTT activated partial thromboplastin time, GCS Glasgow coma scale, COPD chronic obstructive pulmonary disease, CI cerebral infarction, MV mechanical ventilation, ICH intracerebral hemorrhage.

Predictive models for PCS and MCS were developed using XGBoost. Hyperparameter tuning was conducted using a grid search method, with the range and final selection of hyperparameters detailed in Supplementary file 2 Table S3. The final AUROC values for the PCS and MCS models were 0.72 and 0.63, respectively. Additional performance metrics are shown in Table 2. The variable importance ranking generated from the SHAP scores is shown in Fig. 3. Within the PCS prediction model, the variables in descending order of importance are: length of hospital stay, age, activated partial thromboplastin time (APTT), GCS score, comorbid COPD, concurrent cerebral infarction, admission to the surgical department, and mechanical ventilation for more than 7 days (Fig. 3A). For the MCS prediction model, the ranking was: length of hospital stay, mechanical ventilation for more than 7 days, concurrent intracerebral hemorrhage, and sedation for more than 7 days (Fig. 3B). The SHAP summary is shown in Supplementary file 2 Fig. S1.

**Table 2:** Model performance.

Model	Accuracy	Sensitivity	Specificity	Precision	F1 Score	AUROC
**Infection survivors**						
PCS model	0.70	0.73	0.58	0.87	0.79	0.72
MCS model	0.60	0.43	0.81	0.75	0.54	0.63
**Sepsis survivors**						
PCS model	0.73	0.75	0.65	0.87	0.81	0.76
MCS model	0.59	0.68	0.56	0.34	0.47	0.68

Abbreviations: PCS physical component summary, MCS mental component summary, AUROC area under the receiver operating characteristic curves.

### 3.4 Subgroup analysis

**Fig. 4:**
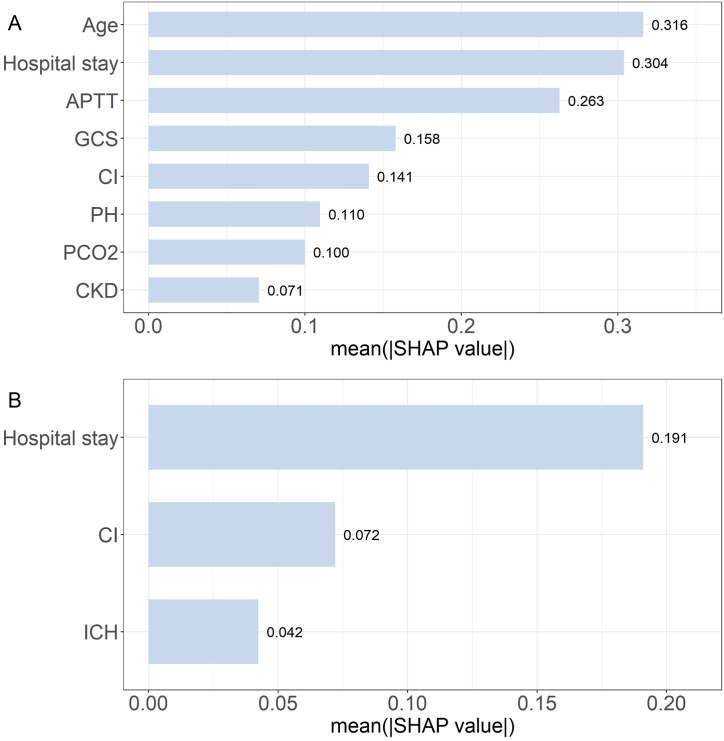
A. Ranking of variable importance for the PCS prediction model in sepsis survivors; B. Ranking of variable importance for the MCS prediction model in sepsis survivors. Abbreviations: PCS physical component summary, MCS mental component summary, APTT activated partial thromboplastin time, GCS Glasgow coma scale, CKD chronic kidney disease, CI cerebral infarction, ICH intracerebral hemorrhage.

A subgroup analysis was performed for patients with a SOFA score of ≥2, which included 756 sepsis survivors with a median follow-up of 514.4 days. Sepsis survivors' scores on all eight dimensions, as well as PCS and MCS scores, were generally lower than the average scores of the general Chinese population (Supplementary file 2 Fig. S2). LASSO regression was used for variable selection (Supplementary file 2 Table S2). Models were developed using XGBoost, with hyperparameter selections detailed in Supplementary file 2 Table S3. The final model performance is shown in Table 2. The AUROC values for the PCS and MCS models were 0.76 and 0.68, respectively. Within the PCS prediction model for sepsis survivors, variables in descending order of importance include: age, length of hospital stay, APTT, GCS score, concurrent cerebral infarction, pH, PCO_2_, and comorbid chronic kidney disease (Fig. 4A). For the MCS prediction model in sepsis survivors, the variables in order of importance were: length of hospital stay, concurrent cerebral infarction, and concurrent intracerebral hemorrhage (Fig. 4B). The SHAP summary is shown in Supplementary file 2 Fig. S3.

## 4 Discussion

In the present study, we identified a pervasive decline in long-term HRQOL for survivors of ICU infections and sepsis, encompassing both psychological and physiological health domains. Subsequently, we developed a predictive model anchored in XGBoost, a sophisticated AI-based machine learning algorithm, to predict long-term HRQOL for these survivors. This AI-enhanced predictive model demonstrated an AUROC of 0.63 for predicting the MCS and an increased AUROC of 0.72 for the PCS. These results underscore the effectiveness of our AI-driven model in predicting long-term HRQOL, although there remains potential for further refinement.

Historically, ICU prediction models have focused primarily on short-term outcomes. Investigations into long-term health outcomes or extended HRQOL have been relatively scarce [32,33]. However, in recent years, as scientific attention to HRQOL has increased, predictive models aimed at long-term HRQOL in ICU patients have emerged. A linear regression model constructed to predict HRQOL in critically ill ICU patients at one year yielded an explained variance (R^2^) of 0.38 [34]. Nonetheless, a predictive model specifically tailored to the long-term HRQOL of ICU patients with infection or sepsis remains uncharted territory.

Our study established a predictive model for long-term HRQOL in ICU survivors of infection or sepsis. This long-term HRQOL prediction model may help clinicians identify high-risk patients earlier, allowing for timely and targeted interventions both before and after discharge to improve long-term outcomes. For instance, targeted bedside rehabilitation therapy in the ICU, which has been shown to improve physical and cognitive function, and post-discharge home rehabilitation, which has been shown to improve HRQOL [35,36]. Additionally, for high-risk patients, personalized post-discharge care plans, such as intensive physical rehabilitation or psychological support, can be formulated. These measures have been proven to improve recovery outcomes and quality of life [37,38].

Our research found that the most important predictor in both models predicting MCS and PCS was length of hospitalization. Numerous studies have found an association between prolonged hospital stays and a decline in HRQOL [15,39]. There is evidence that prolonged bed rest due to prolonged hospitalization may lead to muscle atrophy [40]. An alternative postulation is that prolonged hospitalization precipitates ICU-acquired weakness, a systemic neuromuscular impairment. In the short term, it is strongly correlated with mortality, while its long-term manifestations severely compromise HRQOL [41,42]. Several studies have identified ICU-acquired weakness as an independent determinant of persistent weakness and reduced HRQOL after ICU discharge. Furthermore, predictive factors in our model, including age, sedation, and mechanical ventilation, not only contribute to prolonged hospital stay, but are also independent risk factors for ICU-acquired weakness [42].

A sub-study of the PHANTASi study, which investigated the HRQOL of sepsis survivors, found that patients' PCS was severely impaired 28 days after discharge, while MCS showed relatively milder impairment. Risk factors included prolonged hospital stay, comorbidities, advanced age, and female gender [39]. A Japanese study on post-intensive care syndrome in mechanically ventilated ICU patients showed that 46.9 % of mechanically ventilated patients manifested physical and psychological impairments at the 6-month mark, with PCS and MCS scores worsening by more than 10 points [43]. In addition, a Chinese study focusing on long-term HRQOL in sepsis patients identified older age, female gender, and prolonged mechanical ventilation duration as major risk factors [18]. These findings are consistent with our research results.

Certain complications associated with sepsis may also affect HRQOL. A study evaluating the impact of sepsis-associated disabling conditions (DCs) on HRQOL found that septic patients with concurrent DCs had significantly worse HRQOL than those without DCs [44]. Cerebral impairment often emerges as a risk factor for impaired HRQOL in different patient demographics. Numerous studies have highlighted the influence of factors such as stroke and GCS scores on the long-term prognosis of patients [[45], [46], [47]]. Existing literature confirms that the HRQOL of patients with kidney injury or chronic kidney disease is inferior to that of the general population, even in the absence of concomitant renal failure [48]. In addition, our research uncovered potential long-term HRQOL implications associated with coagulation parameters such as APTT and D-dimer, a facet not previously explored in related predictive models and risk factor analyses. Some research suggests an association between APTT and one-year mortality in septic patients, while another study found that patients with venous thromboembolism, atrial fibrillation, and other comorbidities requiring anticoagulation began to show a substantial decline in quality of life after anticoagulation [49,50]. These complications requiring anticoagulation could potentially worsen HRQOL in their own right.

Our study has several limitations. First, this was a single-center study, and we did not seek data from other sources for external validation. Second, we did not assess patients' initial HRQOL, which could potentially influence the results. Third, a significant proportion of patients were excluded due to loss to follow-up, which could introduce bias and affect the generalizability of the model. Fourth**,** the primary diagnosis of infection was extracted based on ICD coding, which precludes precision regarding the timing of diagnosis, which may introduce bias in the diagnosis of sepsis. Finally, despite the use of LASSO regression, hyperparameter tuning, regularization techniques, and XGBoost's inherent mechanisms to mitigate overfitting or bias in this study, these issues cannot be completely avoided in practical applications. Therefore, future research should focus on external validation to further assess and verify the model's reliability.

## 5 Conclusions

This research successfully formulated an effective predictive model for the long-term HRQOL of ICU patients with infection and sepsis using AI and machine learning techniques, and explored the key factors potentially influencing HRQOL. The model helps clinicians identify high-risk patients, enabling targeted interventions that can improve long-term outcomes. However, the accuracy and applicability of the model require further refinement and validation.

## Funding

This work is supported by 10.13039/501100001809National Natural Science Foundation of China (No. 82072134), the Scientific Research Fund of 10.13039/501100002947Anhui Medical University (No. 2020xkj036), the Key Supporting Discipline of Shanghai 10.13039/100022720Health Bureau (No. 2023ZDFC0104) and the Clinical Key Discipline of Shanghai Pudong Heath Bureau (No. PWYgf2021-03).

## Data availability statement

Publicly available datasets were analyzed in this study. This data can be found here: https://physionet.org/content/icu-infection-zigong-fourth/1.1/.

## Ethics statement

Review and/or approval by an ethics committee was not required for this study because it was an analysis of a third-party, anonymized, publicly available database with pre-existing institutional review board approval.

## Consent for publication

Not applicable.

## CRediT authorship contribution statement

**Yang Zhang:** Writing – original draft, Software, Formal analysis, Conceptualization. **Sinong Pan:** Software, Formal analysis. **Yan Hu:** Formal analysis. **Bingrui Ling:** Methodology, Conceptualization. **Tianfeng Hua:** Writing – review & editing, Supervision. **Lunxian Tang:** Writing – review & editing. **Min Yang:** Writing – review & editing.

## Declaration of competing interest

The authors declare that they have no known competing financial interests or personal relationships that could have appeared to influence the work reported in this paper.

## Abbreviations

ICU: intensive care unit
HRQOL: health-related quality of life
PCS: physical component summary
MCS: mental component summary
LASSO: least absolute shrinkage and selection operator
AI: artificial intelligence
XGBoost: extreme gradient boosting
AUROC: area under the receiver operating characteristic curve
SHAP: shapley additive explanations
EICU: emergency intensive care unit
BP: blood pressure
BUN: blood urea nitrogen
BNP: b-type natriuretic peptide
APTT: activated partial thromboplastin time
INR: international normalized ratio
GCS: Glasgow coma scale
SOFA: sequential organ failure assessment
CHF: congestive heart failure
CKD: chronic kidney disease
COPD: chronic obstructive pulmonary disease
TBI: traumatic brain injury
ICH: intracerebral hemorrhage
MV: mechanical ventilation
DC: disabling condition
