# Supplementary material for: Establishing an artificial intelligence-based predictive model for long-term health-related quality of life for infected patients in the ICU
Source: Heliyon. 2024 Jul 31;10(15):e35521. doi: 10.1016/j.heliyon.2024.e35521 (PMC11336746; doi:10.1016/j.heliyon.2024.e35521)
Supplement: Multimedia component 1 [file mmc1.pdf]

SF-36(tm) Health Survey

Instructions for completing the questionnaire: Please answer every question. Some questions may look like others, but each one is different. Please take the time to read and answer each question carefully by filling in the bubble that best represents your response.

Patient Name: ____

SSN#: ____ Date: ____

Person heling to complete this form: ____

1. In general, would you say your health is:

- ❑ Excellent
- ❑ Very good
- ❑ Good
- ❑ Fair
- ❑ Poor

2. Compared to one year ago, how would you rate your health in general now?

- ❑ Much better now than a year ago
- ❑ Somewhat better now than a year ago
- ❑ About the same as one year ago
- ❑ Somewhat worse now than one year ago
- ❑ Much worse now than one year ago

3. The following items are about activities you might do during a typical day. Does your health now limit you in these activities? If so, how much?

a. Vigorous activities, such as running, lifting heavy objects, participating in strenuous sports.
- ❑ Yes, limited a lot.
- ❑ Yes, limited a little.
- ❑ No, not limited at all.

b. Moderate activities, such as moving a table, pushing a vacuum cleaner, bowling, or playing golf?
- ❑ Yes, limited a lot.
- ❑ Yes, limited a little.
- ❑ No, not limited at all.

c. Lifting or carrying groceries.
- ❑ Yes, limited a lot.
- ❑ Yes, limited a little.
- ❑ No, not limited at all.

d. Climbing several flights of stairs.
- ❑ Yes, limited a lot.
- ❑ Yes, limited a little.
- ❑ No, not limited at all.

e. Climbing one flight of stairs.
- ❑ Yes, limited a lot.
- ❑ Yes, limited a little.
- ❑ No, not limited at all.

f. Bending, kneeling or stooping.
- ❑ Yes, limited a lot.
- ❑ Yes, limited a little.
- ❑ No, not limited at all.

g. Walking more than one mile.
- ❑ Yes, limited a lot.
- ❑ Yes, limited a little.
- ❑ No, not limited at all.

h. Walking several blocks.
- ❑ Yes, limited a lot.
- ❑ Yes, limited a little.
- ❑ No, not limited at all.

i. Walking one block.
- ❑ Yes, limited a lot.
- ❑ Yes, limited a little.
- ❑ No, not limited at all.

j. Bathing or dressing yourself.
- ❑ Yes, limited a lot.
- ❑ Yes, limited a little.
- ❑ No, not limited at all.

4. During the past 4 weeks, have you had any of the following problems with your work or other regular daily activities as a result of your physical health?

a. Cut down the amount of time you spent on work or other activities?
☐ Yes ☐ No

b. Accomplished less than you would like?
☐ Yes ☐ No

c. Were limited in the kind of work or other activities
☐ Yes ☐ No

d. Had difficulty performing the work or other activities (for example, it took extra time)
☐ Yes ☐ No

5. During the past 4 weeks, have you had any of the following problems with your work or other regular daily activities as a result of any emotional problems (such as feeling depressed or anxious)?

a. Cut down the amount of time you spent on work or other activities?
☐ Yes ☐ No

b. Accomplished less than you would like
☐ Yes ☐ No

c. Didn't do work or other activities as carefully as usual
☐ Yes ☐ No

6. During the past 4 weeks, to what extent has your physical health or emotional problems interfered with your normal social activities with family, friends, neighbors, or groups?
- ❑ Not at all
- ❑ Slightly
- ❑ Moderately
- ❑ Quite a bit
- ❑ Extremely

7. How much bodily pain have you had during the past 4 weeks?
- ❑ Not at all
- ❑ Slightly
- ❑ Moderately
- ❑ Quite a bit
- ❑ Extremely

8. During the past 4 weeks, how much did pain interfere with your normal work (including both work outside the home and housework)?

- ❑ Not at all
- ❑ Slightly
- ❑ Moderately
- ❑ Quite a bit
- ❑ Extremely

9. These questions are about how you feel and how things have been with you during the past 4 weeks. For each question, please give the one answer that comes closest to the way you have been feeling. How much of the time during the past 4 weeks.

a. did you feel full of pep?
- ❑ All of the time
- ❑ Most of the time
- ❑ A good bit of the time
- ❑ Some of the time
- ❑ A little of the time
- ❑ None of the time

b. have you been a very nervous person?
- ❑ All of the time
- ❑ Most of the time
- ❑ A good bit of the time
- ❑ Some of the time
- ❑ A little of the time
- ❑ None of the time

c. have you felt so down in the dumps nothing could cheer you up?
- ❑ All of the time
- ❑ Most of the time
- ❑ A good bit of the time
- ❑ Some of the time
- ❑ A little of the time
- ❑ None of the time

d. have you felt calm and peaceful?
- ❑ All of the time
- ❑ Most of the time
- ❑ A good bit of the time
- ❑ Some of the time
- ❑ A little of the time
- ❑ None of the time

e. did you have a lot of energy?
- ❑ All of the time
- ❑ Most of the time
- ❑ A good bit of the time
- ❑ Some of the time
- ❑ A little of the time
- ❑ None of the time

f. have you felt downhearted and blue?
- ❑ All of the time
- ❑ Most of the time
- ❑ A good bit of the time
- ❑ Some of the time
- ❑ A little of the time
- ❑ None of the time

g. did you feel worn out?
- ❑ All of the time
- ❑ Most of the time
- ❑ A good bit of the time
- ❑ Some of the time
- ❑ A little of the time
- ❑ None of the time

h. have you been a happy person?
- ❑ All of the time
- ❑ Most of the time
- ❑ A good bit of the time
- ❑ Some of the time
- ❑ A little of the time
- ❑ None of the time

i. did you feel tired?
- ❑ All of the time
- ❑ Most of the time
- ❑ A good bit of the time
- ❑ Some of the time
- ❑ A little of the time
- ❑ None of the time

10. During the past 4 weeks, how much of the time has your physical health or emotional problems interfered with your social activities (like visiting friends, relatives, etc.)?
- ❑ All of the time
- ❑ Most of the time
- ❑ Some of the time
- ❑ A little of the time
- ❑ None of the time

11. How TRUE or FALSE is each of the following statements for you?

a. I seem to get sick a little easier than other people
- ❑ Definitely true
- ❑ Mostly true
- ❑ Don't know
- ❑ Mostly false
- ❑ Definitely false

b. I am as healthy as anybody I know
- ❑ Definitely true
- ❑ Mostly true
- ❑ Don't know
- ❑ Mostly false
- ❑ Definitely false

c. I expect my health to get worse
- ❑ Definitely true
- ❑ Mostly true
- ❑ Don't know
- ❑ Mostly false
- ❑ Definitely false

d. My health is excellent
- ❑ Definitely true
- ❑ Mostly true
- ❑ Don't know
- ❑ Mostly false
- ❑ Definitely false
