# Supplementary material for: Establishing an artificial intelligence-based predictive model for long-term health-related quality of life for infected patients in the ICU
Source: Heliyon. 2024 Jul 31;10(15):e35521. doi: 10.1016/j.heliyon.2024.e35521 (PMC11336746; doi:10.1016/j.heliyon.2024.e35521)
Supplement: Multimedia component 2 [file mmc2.pdf]

Establishing an artificial intelligence-based predictive model for long-term health-related quality of life for infected patients in the ICU

Yang Zhang [a, b], Sinong Pan [a, b], Yan Hu [a, b], Bingrui Ling [a, b], Tianfeng Hua [a, b], Lunxian Tang [c, *], Min Yang [a, b, *]

[a] The Second Department of Critical Care Medicine, The Second Affiliated Hospital of Anhui Medical University, Hefei, Anhui 230601, P. R. China

[b] Laboratory of Cardiopulmonary Resuscitation and Critical Care, The Second Affiliated Hospital of Anhui Medical University, Hefei, Anhui 230601, P. R. China

[c] Department of Internal Emergency Medicine (North), Shanghai East Hospital, Tongji University School of Medicine, Shanghai 200120, P. R. China

* Corresponding author at: The Second Department of Critical Care Medicine, The Second Affiliated Hospital of Anhui Medical University, 678 Furong Road, Hefei, Anhui 230601, P. R. China (M. Yang). Department of Internal Emergency Medicine (North), Shanghai East Hospital, Tongji University School of Medicine, 551 South Pudong Road, Shanghai 200120, P. R. China (LX. Tang).

Email addresses: yangmin@ahmu.edu.cn (M. Yang), 456tlx@163.com (LX. Tang).

Table S1 Variables selected by Lasso regression and coefficients of variables (β)

Variables	Coefficient (β)	Variables	Coefficient (β)
**PCS model**			
Age	1.685077×10^{-2}	CI	1.308592×10^{-1}
Hospital stay	9.455457×10^{-5}	MV>7d	2.454631×10^{-1}
APTT	-1.707187×10^{-3}	Admission Other	-3.054713×10^{-1}
GCS	-2.679518×10^{-2}	Infection Digestive	-8.541652×10^{-3}
COPD	9.268087×10^{-2}	Discharge Other	-3.332025×10^{-5}
Hypertension	1.671737×10^{-2}		
**MCS model**			
Hospital stay	2.653125×10^{-6}	Sedation>7d	2.249478×10^{-2}
ICH	1.276785×10^{-1}	MV>7d	1.292916×10^{-1}

Abbreviations: APTT activated partial thromboplastin time, GCS glasgow coma scale, COPD chronic obstructive pulmonary disease, ICH intracerebral hemorrhage, CI cerebral infarction, MV mechanical ventilation.

Table S2 Variables selected by Lasso regression and coefficients of variables (β) in subgroup analysis

**Variables**	**Coefficient (β)**	**Variables**	**Coefficient (β)**
**PCS model**			
Age	1.513353×10^{-2}	ICH	9.311827×10^{-2}
Hospital stay	9.502553×10^{-5}	CI	3.022355×10^{-1}
APTT	-1.637681×10^{-2}	MV>7d	1.866330×10^{-1}
pH	-2.904246×10^{-1}	Admission Other	-6.543114×10^{-1}
PCO_2	2.522439×10^{-3}	Admission Surgical	-5.652762×10^{-2}
GCS	-3.137961×10^{-2}	Infection Digestive	-3.019724×10^{-1}
CKD	5.539421×10^{-1}	Discharge Other	-2.957819×10^{-2}
COPD	1.530357×10^{-1}	Discharge Surgical	-1.137273×10^{-5}
**MCS model**			
Hospital stay	2.304322×10^{-5}	CI	5.215756×10^{-2}
ICH	3.606004×10^{-1}		

Abbreviations: APTT activated partial thromboplastin time, GCS Glasgow coma scale, CKD chronic kidney disease, COPD chronic obstructive pulmonary disease, ICH intracerebral hemorrhage, CI cerebral infarction, MV mechanical ventilation.

Table S3 Hyperparameter search space and final selected values for PCS and MCS models

Hyperparameter	Search Space	Chosen Hyperparameter Value			
		Infection survivors		Sepsis survivors	
		PCS model	MCS model	PCS model	MCS model
max_depth	[2,3,4,5]	2	3	2	3
eta	[0.01,0.1,0.3]	0.1	0.01	0.1	0.01
gamma	[0.25,0.5]	0.25	0.25	0.25	0.25
nrounds	[75,100,125]	75	75	75	75

max_depth: maximum depth of a tree, eta: learning rate, gamma: minimum loss reduction required, nrounds: number of boosting iterations.

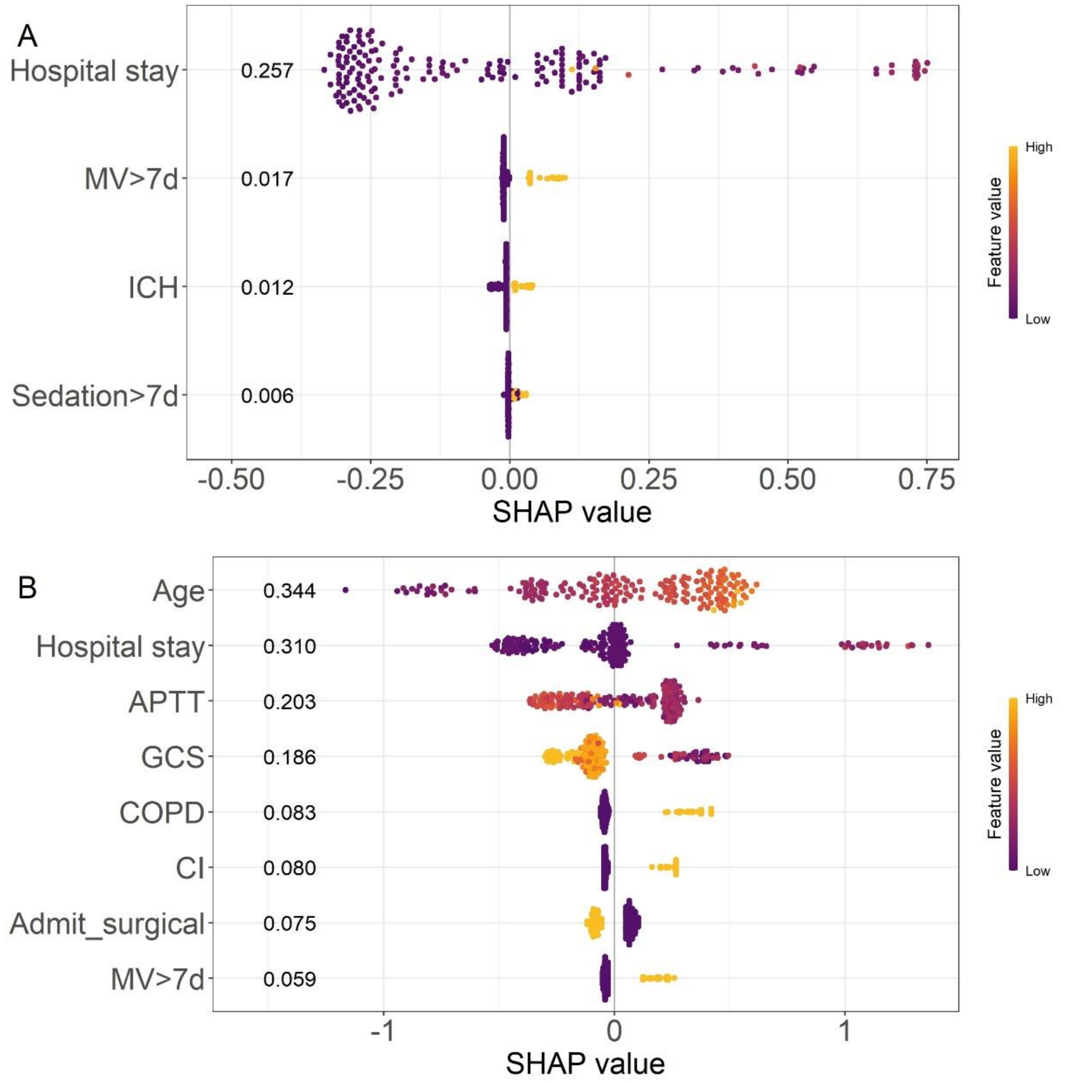

Fig. S1. A. SHAP summary plot of the PCS prediction model; B. SHAP summary plot of the MCS prediction model.

Abbreviations: PCS physical component summary, MCS mental component summary, APTT activated partial thromboplastin time, GCS glasgow coma scale, COPD chronic obstructive pulmonary disease, CI cerebral infarction, MV mechanical ventilation, ICH intracerebral hemorrhage.

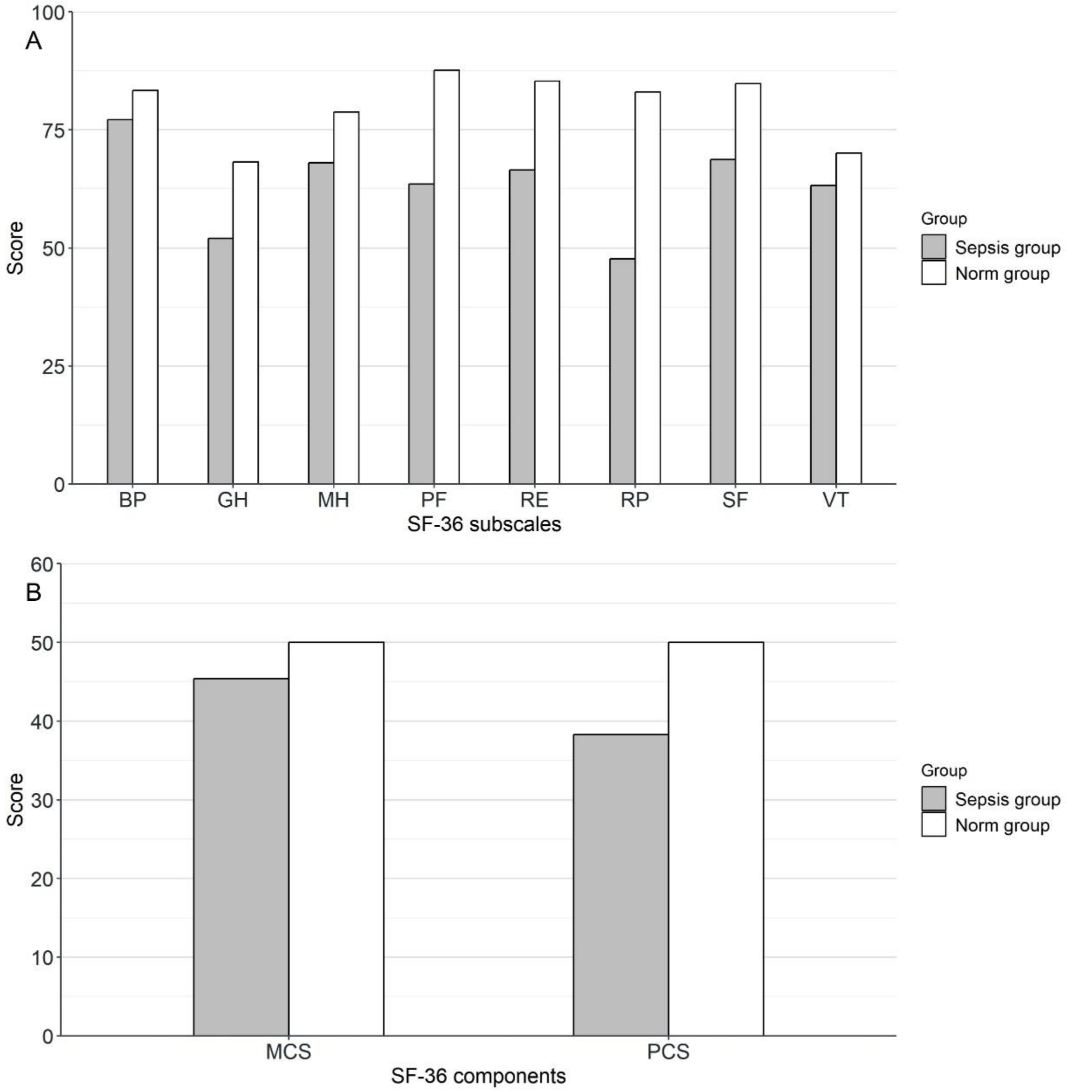

Fig. S2. A. Comparison of mean scores for the eight dimensions of the SF-36 scale between ICU sepsis survivors and Chinese standard norms; B. Comparison of mean MCS and PCS scores between ICU sepsis survivors and Chinese standard norms.

Abbreviations: PF physical functioning, RP role-functioning physical, BP bodily pain, GH general health, VT vitality; SF social functioning, RE role-functioning emotional, MH mental health, PCS physical component summary, MCS mental component summary.

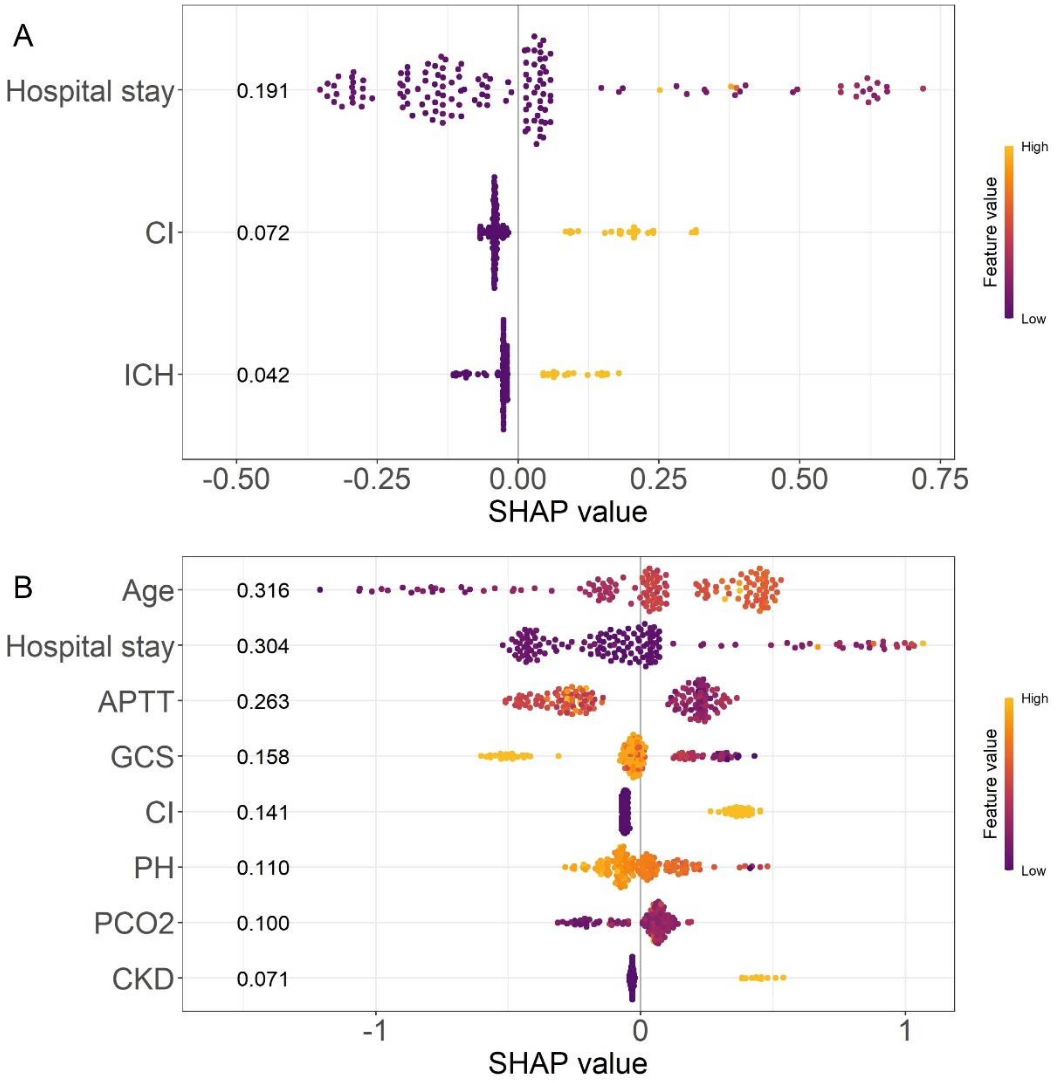

Fig. S3. A. SHAP summary plot of the PCS prediction model for sepsis survivors; B. SHAP summary plot of the MCS prediction model for sepsis survivors.

Abbreviations: PCS physical component summary, MCS mental component summary, APTT activated partial thromboplastin time, GCS Glasgow coma scale, CKD chronic kidney disease, CI cerebral infarction, ICH intracerebral hemorrhage.
